# Equity of access under Korean national long-term care insurance: implications for long-term care reform

**DOI:** 10.1186/s12939-015-0210-y

**Published:** 2015-09-15

**Authors:** Ju Moon Park

**Affiliations:** Department of Urban Policy and Administration, Incheon National University, 119 Academy-ro, Yeonsu-gu, Incheon, 402-750 South Korea

## Abstract

**Background:**

The national long-term care insurance was implemented in July 2008. Few studies have been conducted with representative national survey data since the long-term care insurance was introduced. Therefore, this study examines the extent to which equity in the use of long-term care has been achieved in Korea.

**Methods:**

The Aday-Andersen model was used as a conceptual model, based on the Korean Health Panel Study which was conducted in 2011. Descriptive and logistic regression analysis was performed to examine the relationship between the dependent and independent variables and the relative importance of factors as predictors of utilization.

**Results:**

The results of this study indicated that those who rated his or her health to be fair, good, and very good, had no limited activities, were disabled, and had insurance coverage were more likely to use long-term care services, respectively. Their decision to use long-term care was primarily affected by need (health status, limited activity, disability) and enabling (insurance coverage) factors. The findings also indicated that the introduction of a national long-term care insurance program did not yield a fully equitable distribution of services.

**Conclusions:**

Long-term care reforms in Korea should continue to concentrate on expanding insurance coverage and reducing the inequities reflected in disparities in consumer cost-sharing and associated patterns of utilization across plans. The subsequent impact on managed care and expenditures need to be more fully understood.

## Background

The Republic of Korea (hereafter Korea) introduced universal health insurance coverage in 1989, through a combined publicly and privately financed national health insurance system. The goal of full coverage was realized with a dual system of compulsory wage-based insurance for workers in the private sector, government employees, and the self-employed and a government-financed program of medical assistance for the poor [1]. While health insurance coverage includes outpatient care, inpatient care, and prescription pharmaceuticals, no coverage for long-term care was included. In response to this, and due to the demographic and cultural changes affecting the need and provision of long-term care, the national long-term care insurance was implemented in July 2008 [2].

The Korean long-term care insurance covers two categories of service benefits: home care and institutional care [3–5]. Home care services include home visiting care, home bathing, home nursing, day and night care, short-term respite care, and others. Institutional care services include a set of long-term nursing care and rehabilitation services. It is possible to choose between institutional care and home-care if one is eligible for long-term care insurance benefits. The long-term care insurance provides coverage for individuals aged 65 or older and those below 65 with debilitating conditions, along with eligibility test through the national care need-assessment system [3]. Those younger than 65 years of age suffering from a senile disease may be recognized as those requiring long-term care, and they accounted for 7.4 % of persons in need of long-term care as of June 2011 [6, 7]. In order to be eligible for the long-term care insurance benefit, the insured persons request a care needs assessment from the National Health Insurance Corporation (NHIC). The care needs of all applicants for long-term care insurance benefits are evaluated using a 52-item screening tool and then, after the assessment, care needs are classified into one of six grades – from one, very urgent, to six, near to normal. Those^1^ whose care needs are from one (Grade I) to three (Grade III) are entitled to the long-term care insurance benefit [8]. Eligibility for long-term care insurance should be revaluated once a year, in principle.

Long-term care insurance is financed by the government (20 %), copayments (up to 20 %), and insurance contributions (60–65 %). The copayment for home care services is 15 %, while that of institutional care is 20 %. However, the poor are exempt from copayments, and individuals^2^ with certain conditions face reduced copayments.

The number of eligible beneficiaries has consistently increased since the introduction of the long-term care insurance, from 146,643 persons in July 2008 to 341,788 persons in December 2012 [5, 7]. The Korean National Health Insurance Corporation (2014) reported that 88.5 % of the respondents were satisfied with the services under the long-term care insurance. Several issues, however, should be addressed for further improvement of the Korean long-term care insurance, including limited coverage and disparities in use [2, 8]. Several studies have been conducted on long-term care utilization in Korea; however, most of them were before the inception of long-term care insurance in 2008, on the intention to utilize a care, and a few were conducted with a sample from a demonstration long-term care insurance program [2, 9–12] or with a sample of actual long-term care insurance beneficiaries [3] or with unrepresentative survey data [13]. Few studies have been conducted with representative national survey data since the long-term care insurance was introduced.

This study examines the extent to which equity in the use of long-term care has been achieved in Korea. The findings are based on the data from the 2011 Korea Health Panel Survey (KHPS). The Aday-Andersen behavioral model is used to guide empirical and normative assessment of equity within the Korean long-term care system. Unlike other utilization models, the model is appropriate for understanding differential levels of access among policy-relevant subgroups and suggesting ways to achieve equity among them [10]. The model coincides best with the aim of this study.

Two questions with respect to equity of access in the use of long-term care services are addressed: *(a)* which subgroups of the Korean population are most likely to have utilized long-term care services, and *(b)* to what extent are the subgroup differences in utilization related to need? This study hypothesizes that the Korean long-term care system will be equitable.

## Methods

### Conceptual model

The Aday and Andersen model [14–21] is used to guide the analyses. In this framework, a series of predisposing, enabling, and need factors are hypothesized to be predictive of utilization of services. The predisposing component includes those variables that describe the “propensity” of individuals to use services. The enabling component describes the “means” individuals have available to them for the use of services. The need component refers to the illness level, which is the most immediate cause of long-term care utilization [18].

Equity of access to long-term care is measured based on the relative importance of need compared to other determinants of long-term care utilization. Access is equitable to the extent that predisposing, need-related demographic factors such as age and sex, as well as illness, account for long-term care utilization. Inequity is, however, suggested if services appear to be distributed on the basis of other predisposing, enabling variables, rather than need.

**Fig. 1:**
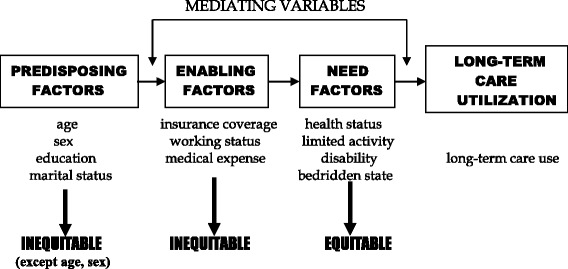
Conceptual framework for this study. The enabling and need factors are mediating variables that help to explain differences between subgroups that might be due to either equitable (need) or inequitable (enabling) factors. Age and sex serve as proxies for need because of the well-established relationships between illness patterns and age and sex. Other predisposing variables are, however, inequitable factors

The analyses will focus on subgroup differences in whether an individual used long-term care services in the one year preceding the interview, and a systematic series of multivariate (logistic regression) analyses examining the extent to which these differences are explained by equitable (need-related) or inequitable (non-need-related) factors (Fig. 1).

### Study sample

The present study focuses on cross-sectional analyses, using the KHPS, which was released to the public in 2014. Baseline data were collected between May 12 and December 10, 2011. Through a face-to-face interview survey, the KHPS provides information on demographic characteristics, service utilization behavior, medical expenditure and health behaviors of the targeted households and their members. With respect to the long-term care insurance a total of 2, 853 individuals were surveyed. The sampling frame for the KHPS Household Component was drawn from respondents in accordance with the 2010 National Population and Housing Census. KHPS sampling weights incorporated adjustment for the complex sample design and reflected survey nonresponse and population totals from the current population survey; weights were applied in all statistical analyses to obtain nationally representative estimates. The KHPS Review Board granted an exemption for this research.

### Measures

The predisposing and enabling measures included age, gender, education, marital status, medical expense, health insurance, and working status. The need and health-related measures included health status, bedridden state, restricted activity, and disability. The dependent variable is a dichotomy reflecting whether or not an older person used any type of long-term care (yes or no).

There are no or minimal missing information on all predisposing, enabling, and need variables corresponding to the Aday and Andersen model with the following exceptions. Many individuals have not evaluated their health status (*n* = 212 or 7.4 % of the sample). There were 45 (or one and a half percent) of the cases for which data on a bedridden state was missing. Restricted activity was also missing in 45 cases.

For the logistic regression analyses, the independent variables were re-coded to indicate dichotomies. The first category for a variable was coded 1 and the reference category for it (after “vs.”) was coded zero.

The several measures of health status were all reported in an interview format and range from highly subjective (e.g., self-evaluation) to more objective (e.g., conditions checked by a nursing assistant) [16]. In this study, four measures of health status and need were respectively represented as a dichotomy, i.e., (i) a self-evaluation of health as ‘fair/good/very good’ (1) versus ‘poor/very poor’ (0), (ii) being a bedridden state as ‘yes’ (1) versus ‘no’ (0), (iii) having a restricted activity as ‘yes’ (1) versus ‘no’ (0), and (iv) having a disability as ‘yes’ (1) versus ‘no’ (0). In addition to the health status of the elderly, the utilization of long-term care services is closely related to demographic factors – in particular age and sex. As proxies for need, age and sex were respectively represented as a dichotomy, i.e., ‘65–74’ (1) versus’75 and older’ (0) and ‘male’ (1) versus ‘female’ (0) in this analysis. Marital status was used in this analysis and was represented by the binary variable ‘married’ (1) versus ‘unmarried or separated or divorced or widowed’ (0). A categorical factor indicating the level of education attained was used as a supplementary measure of socio-economic status. In this study, education was represented by the binary factor ‘no schooling’ (1) versus ‘primary schooling or more’ (0).

The enabling variables chosen for this analysis included medical expense, health insurance, and working status. In the present analysis, medical expense refers to the total medical expense respondents or their family members spent during the twelve months preceding the interview. The medical expense was represented by the dichotomous factor ‘>7,000,000 won’ (1) versus ‘0-7,000,000 won’ (0). As a resource variable, having health insurance is closely related with long-term care utilization. In this study, health insurance is determined by the following question: “What type of health plan do you have?” Health plan refers to insurance coverage the study subject used. It was categorized in four categories, i.e., ‘government plan’, ‘vocational plan’, ‘regional plan’, and ‘medical aid’. Insurance coverage is represented as a dichotomy, i.e., ‘medical aid’ (1) versus ‘other insurance coverage [health plan for employees of the government, private school, and industrial establishments and health plan for the self-employed’ (0). Working status refers to participating in labor force. It is represented as the dichotomous factor, ‘yes’ (1) versus ‘no’ (0).

### Data analysis

Descriptive statistics such as mean, standard deviation (SD), frequency and percentage were used to analyze the individual characteristics of the sample. A series of bivariate analyses and associated chi-squares were performed to examine the relationship between each of the predisposing, enabling and need variables and long-term care utilization. Logistic regression analysis was then used to examine the relative importance of factors found to be significant in the bivariate analyses in predicting whether or not an individual used long-term care services.

A systematic series of multivariate (logistic regression) analyses were conducted to address the relative importance of the respective predisposing, enabling and need factors as predictors of utilization. The predisposing variables were entered in stage 1 to examine demographic subgroup differences. The need variables were entered in stage 2 to examine the extent to which subgroup differences in stage 2 were reduced when variations in the need for care were controlled. At the final stage, the enabling factors were entered to examine whether remaining subgroup differences were due primarily to the availability of personal or medical care resources (stage 3).

A logistic regression is useful for estimating models reflecting the relationship between the dependent variable and the independent variables, when the dependent variable is binary or dichotomous. Also, the application of a logistic regression to this analysis is appropriate because of the small number of cases that did not utilize long-term care services which resulted in the skewness of the principal dependent variable.

The statistical significance of the odds ratios (the ratio of the likelihood that one age group, e.g., 65–74 years, has access compared to another age group, e.g., 75 + years) was examined to evaluate the impact of the predisposing, enabling, and need factors at each stage. Changes in the magnitude or significance of the odds ratios in the successive stages were used to identify those factors that might help to account for subgroup differences in the probability of using long-term care services. An equitable distribution of services would be reflected in demographic subgroup (except for age and sex) differences (stage 1) being largely explained by differences in need (stage 2). Empirically, this effect would be documented by the odds ratio becoming non-significant or remaining significant (*p* ≤ 0.05) but increasing (>) or decreasing (<) substantially (10+ %) in stage 2. An inequitable distribution of services would be reflected in the extent to which resource factors (such as health plan, working status, and medical expense) have strongly independent or explanatory effects in accounting for variations in use (stage 3). Empirically, significant odds ratios for these factors in the stage 3 analyses would document their independent effects in predicting long-term care utilization. Substantial changes in the odds ratios for other variables (increasing or decreasing 10+ % or becoming non-significant) from stage 2 to stage 3 of the analyses, would attest to the explanatory effects of the enabling variables, that is, they help to account for (or explain) differences between demographic subgroups. In either case, the findings point to variations in use due to the availability of these resources.

The −2 x (log likelihood) test, based on the chi-square distribution, was used to test the maximum likelihood fit of the model, that is, the extent to which the probabilities of occurrences predicted by the model were an accurate representation of the actual occurrences (such as, whether or not long-term care was used).

## Results

### Sample characteristics

The predisposing, enabling, and need characteristics along with long-term care utilization are presented in Table 1. Survey respondents had a higher percentage with primary schooling, a higher percentage with vocational plan, and a higher percentage of those who were female, unemployed, and married (Table 1). The average age of the respondents was 70.9 ± 5.7 years old. 56.3 % of the respondents were female. 66.7 % of the respondents had a spouse. 17.9 % of the respondents had no schooling; 44.0 % had primary schooling; 38.1 % had middle schooling or higher. 8.1 % of the respondents had government plan; 52.5 % had vocational plan; 28.4 % had regional plan; 11.0 % had medical aid. The medical expense of the respondents ranged from 0 to 22,927,398 won ($US 19,940) and the yearly average medical expense respondents or their family members spent was 947,090 won. 33.4 % of the respondents were employed. 28.8 % of the respondents evaluated their health as poor or very poor; 71.2 % evaluated their health as one of fair, good, and very good. 14.0 % of the respondents had a limited activity; 83.0 % had a disability; 8.1 % were in a bedridden state.

**Table 1:** Characteristics of respondents (*n* = 2,853)

Study variables	Value
Predisposing characteristics	
Age, years [mean (±SD)]	70.9 (±5.7)
Female, %	56.3
Education (graduation), %	
No schooling	17.9
Primary schooling	44.0
Middle schooling or more	38.1
Having a spouse, %	66.7
Enabling characteristics	
Having working status, %	33.4
Type of health plan, %	
Government plan	8.1
Vocational plan	52.5
Regional plan	28.4
Medical aid	11.0
Family medical expense, 1,000won^#^ [mean (±SD)]	947.1 (±1,608.1)
Health needs	
Self-rated health, %	
Fair+	71.2
Poor/very poor	28.8
Having limited activity, %	14.0
Being in a bedridden state, %	8.1
Having a disability, %	83.0

^#^Korean monetary unit ($US 1 = KRW 1,150)

### Bivariate analysis

Those who were most likely to have used long-term care services included those who were 65–74 years old, those who were married, those who were uneducated, those who were employed, those who had regional plan, those who spent the medical expense of >7,000,000 won, those who rated their health as fair or good or very good, those who had no limited activities, those who were in a bedridden state, and those who had a disability (Table 2).

**Table 2:** Percentage of those who used long-term care services by each study variable (*N* = 2,853)

Study variables	%	*χ* ^2^
Predisposing		
Age (years)		71.5^a^
65–74	1.3	
75–84	1.2	
85+	0.6	
Sex		0.4
Male	1.3	
Female	1.9	
Education		23.1^a^
No schooling	1.2	
Primary schooling	1.1	
Middle schooling or more	0.9	
Marital status		13.9^a^
Married	1.6	
Others	1.5	
Enabling		
Working status		43.1^a^
Yes	3.1	
No	0.0	
Type of health plan		31.2^a^
Government plan	0.9	
Vocational plan	0.7	
Regional plan	1.3	
Medical aid	0.2	
Family medical expense		13.4^a^
> 7,000,000 won	2.9	
≤ 7,000,000 won	0.2	
Need		
Health status		117.9^a^
Fair+	1.1	
Poor/very poor	0.1	
Limited activity		234.7^a^
Yes	0.4	
No	1.8	
Bedridden state		117.9^a^
Yes	1.2	
No	1.0	
Disability		99.9^a^
Yes	1.8	
No	1.4	

^a^ *P* < 0.01

Note: The number of cases on which the estimates are based is 2,853, except for the following variables (for which the number of cases is noted in parentheses): health status (2,641), bedridden state (2,808), and limited activity (2,808)

In the initial stage of the analyses, the variables such as age, education, marital status, working status, health insurance, medical expense, self-perceived health status, limited activities, bedridden state, and disability remained significant predictors of long-term care utilization. All tests were conducted at the 5 % level of significance.

### Multivariate analysis

The odds ratios for long-term care utilization, simultaneously adjusted for multiple independent variables, are presented in Table 3. After adjusting for an array of predisposing factors (stage 1), older adults who were most likely to have used long-term care services included those aged 65 to 74, those who have primary schooling or more, and those who were married. Among all the predisposing variables, three variables, i.e., age, education, and marital status (except for sex) were significantly associated with whether or not long-term care was utilized.

**Table 3:** Multivariate logistic regression analysis of predictors of long-term care utilization in last twelve months--Korea, weighted (2011)

	Long-term care utilization					
Determinants	Stage I		Stage II		Stage III	
	OR	*p*	OR	*p*	OR	*p*
Predisposing						
Age (years)						
65–75 vs 75+	3.16	<0.01	1.28	0.65	0.95	0.93
Sex						
Male vs female	6.52	0.13	1.07	0.88	1.14	0.78
Education						
No schooling vs primary schooling or more	0.51	<0.01	0.93	0.88	0.97	0.95
Marital status						
Married vs others	1.72	<0.05	1.42	0.43	1.27	0.57
Need						
Health status						
Fair + vs poor/very poor			15.77	<0.01	13.24	<0.01
Limited activity						
Yes vs no			0.22	<0.01	0.24	<0.01
Bedridden state						
Yes vs no			0.47	0.07	0.58	0.23
Disability						
Yes vs no			4.02	<0.01	3.1	<0.01
Enabling						
Working status						
Yes vs no					6.55	0.07
Type of health plan						
Medical aid vs others					0.45	<0.05
Family medical expense						
> 7,000,000 won vs ≤7,000,000 won					0.48	0.42
Model *χ* ^2^	48.08		101.48		111.77	
Degree of freedom	4		8		11	
Significance	<0.0001		<0.0001		<0.0001	

These relationships were re-examined, adjusting for need (stage 2). The elderly with fair or good or excellent health status were more likely to have used long-term care services than those with poor or very poor health status. Older Koreans who had a limited activity were much less likely to have used long-term care services than their counterparts. Those who had a disability were much more likely to have used long-term care services than older persons who had no disability.

All the need variables selected in this study had a notable impact on the odds ratios of long-term care utilization for the predisposing variables entered in stage 2. All the demographic subgroup differences were in general substantially narrowed in stage 2, that is, the odds ratios shifted toward unity. Specifically, the variables indicating those aged 65 to 74 versus those over 75 years of age, education, and marital status, became non-significant at the 0.05 level. The findings suggest that the need factors related to health status, disability, and limited activity remain important predictors of the use of long-term care among older Koreans (stage 2).

The impact of the enabling factors was examined in stage 3. The enabling factors were working status, insurance coverage, and medical expense. Those who had a health plan were much more likely to have used long-term care services than those who had medical aid. Adjusting for having a health plan had little impact on the odds ratios of long-term care utilization for the predisposing and need factors. The remaining subgroup differences remained about the same once the resource variables were taken into account.

In sum, having a health plan did not fully ameliorate the remaining subgroup differences in the use of long-term care services among older Koreans, observed in stage 3. Nonetheless, having a health plan remain significant independent determinants of long-term care utilization.

The chi-square based test for assessing how well the models fit the data was significant, i.e., *p* < 0.0001 (Table 3).

## Discussion

Given the rapidly growing older population in Korea and the increasing number of long-term care beneficiaries, more effectively targeting populations at risk is an essential part of both improving services to older adults and reducing disparities in care. Identifying the demographic subgroups that are least likely to use long-term care services is a first step in developing targeted health care interventions. Yet little work has focused on identifying how equitable the population subgroup differences might be. The research reported here addresses this issue.

The results of this study do not fully support the hypothesis that the Korean long-term care insurance system is equitable. In the multivariate analysis, this study reveals that older Koreans’ health status and need are important determinants of whether or not they have used long-term care services. Differences in need substantially account for the original differences observed between subgroups of older Koreans (see Table 3). The results also establish that having insurance coverage has little impact on the odds ratios of long-term care utilization for subgroups of older Koreans. The remaining subgroup differences remained about the same once the resource variables are taken into account. Nonetheless, insurance coverage remains important independent predictor of access.

The Korean long-term care insurance program does not yield a fully equitable distribution of services for older Koreans, who were reported in the existing long-term care literature as a group with higher needs, but limited access to care [14, 18]. Possible explanations for inequalities are the fact that certain subgroups, i.e., those with medical aid coverage were less likely to have used long-term care services than the self-employed, and employees of the government, private schools, and industrial establishments to use long-term care services, even after the other factors were taken into account, but there may have been other explanations that were not evident.

The data suggest that a national long-term care system exists in Korea with access problems for people covered in some of the health plans. Older Koreans with medical aid^3^ have the heavy cost-sharing burden comparable to that paid by the patients with health plans. Especially, for Type 2 beneficiaries, a higher burden of copayment leads to limited financial protection, and this can become a barrier to long-term care utilization, which results in inequity and differential long-term care utilization across different socio-economic groups [22]. However, a subsidy for copayment may result in facilitating a higher level of long-term care utilization for Type 1 beneficiaries. Moreover, such a higher utilization of long-term care by Type 1 beneficiaries is possible as the Korean long-term care system has no gate-keeping or managed care. To curb the increasing spending, governments have recently introduced managed care to monitor and guide medical aid beneficiaries with a higher need of health care [23-26], but its effectiveness is still under evaluation. Considering the fact that individuals are already responsible for food and extensive service cost, a 20 % co-payment appears to be too high compared to the 10 % co-payment of Japan [27]. The findings that older Koreans with medical aid are less likely than their counterparts to use long-term care services provide evidence that they do not have full financial access to long-term care services under the current system.

This study contributes to the existing literature on long-term care equity: as far as I know, this study is the first study to examine the extent to which equity in the use of long-term care services has been achieved in Korea, using national long-term care survey data. There is literature on long-term care equity in general. For example, Theobald [26] has shown that German long-term care insurance, despite its universalism and social rights basis, has led to inequity in assessment and care situations, depending on gender, living arrangements, family status, socioeconomic status, and ethnicity. Rodrigues and Schmidt [28] have shown that different systems and policies have different implications for whether high- or low-income groups benefit from formal home care services. Kim and others [3] have shown that the subsidy policy in Korea positively contributes to equity in access to long-term care. Few studies, however, have evaluated the equity of the Korean long-term care system. Kim and others, as mentioned above, examined the impact of the subsidy policy on long-term care utilization, like Sato et al.’s study [29] on the policy’s impacts on the long-term care insurance program in Japan. Methodologically, unlike Kim et al. [3], who analyzed secondary administrative data, this study analyzed national long-term care survey data. There are also several limitations. The model was limited to the data collected by the Korea Health Panel Study in 2011. There is a difficulty that arises as the result of using the model with the secondary data, e.g., as for the study design, none of the established association can be inferred as a cause-effect relation. Because of small sample size, this analysis did not focus on using institutional care vs. home care among the users, but on using any type of long-term care (yes or no). Also, the data did not include some independent variables (e.g., income and residence) that might affect long-term care utilization. In previous studies, income and residence were found to influence long-term care utilization [3, 13]. There could also be unobserved factors associated with long-term care utilization in this study due to the limitation of the data. These variables should be included in future research. Finally, the long (one-year) recall period used to ask about long-term care utilization can increase the amount of bias associated with respondent memory loss. Although the U.S. National Center for Health Statistics National Health Interview Survey (NCHS-NHIS) uses a two-week recall period, it is a study that is continuously in the field throughout the year, and the data is used to construct aggregate estimates of the volume of visits for the U.S. population [30]. In contrast, the KHPS uses a one-year recall period and the data is used to construct the volume of visits estimates for individuals. Therefore, seasonal bias and random errors are more likely to be problematic with the latter approach.

## Conclusions

This study provides evidence that the introduction of a national long-term care insurance program does not yield a fully equitable distribution of services. At the same time, this study suggests that two main policy implications for long-term care reform in Korea may be drawn from the findings reported upon here: *(a)* a “universal” national insurance plan does not insure that everyone has access to care, and *(b)* a mixed public and private system is likely to produce wide variability in both the scope of plan benefits and the burden of consumer cost-sharing.

Even with universal health insurance, some vulnerable populations (e.g., those with medical aid coverage) still do not have access to care [1, 31]. This can happen in one of two ways – either access to general long-term care remains inaccessible or non-insured services such as food and extensive services are not covered under universal health insurance.

Persons with different types of insurance coverage differ in their use of long-term care. Older Koreans with medical aid are less likely than their counterparts to use long-term care services, which may be due to the heavy cost-sharing burden (premiums, deductibles, and co-insurance) that members of individual medical aid programs are more likely to bear [1].

Variations in the patterns of use of long-term care for those with medical aid point to the fact that non-financial policy options or modifications of the existing financing system may be required to enhance access for these groups.

In sum, this study has suggested that long-term care reforms in Korea should continue to concentrate on expanding insurance coverage and reducing the inequities reflected in disparities in consumer cost-sharing and associated patterns of utilization across plans. The subsequent impact on managed care and expenditures need to be more fully understood. In addition, further research is needed to identify the nonfinancial barriers that persist for those with medical aid coverage.

## Consent

Written informed consent was obtained from the patient for the publication of this report.

## Footnotes

1 The grade is determined on the basis of the number of points of the need for long-term care. The number of points required for recognition of the need for long-term care is 95 or more for Grade I, 75 to 94 for Grade II and 51 to74 for Grade III.

2 Individuals with certain condition include the disabled, people with rare and incurable diseases, and the marginally poor.

3 It is divided into Type 1 medical aid and Type 2 medical aid. Type 1 medical aid beneficiaries (57 %) do not pay cost sharing, while type 2 (43 %) beneficiaries pay the cost sharing comparable to that paid by the patients of (social) health insurance program.

## References

[1] Park JM (2015). Equity of access under Korean universal health insurance. Asia Pac J Public Health.

[2] Kim H, Lim W. Formal long-term care subsidies, informal care, and medical expenditures. 2012. www.columbia.edu/.../ltc_for_publication_v04.pdf. Accessed 20 March 2015.

[3] Kim H, Kwon S, Yoon NH, Hyun KR (2013). Utilization of long-term care services under the public long-term care insurance program in Korea: implications of a subsidy policy. Health Policy..

[4] Help wanted? Providing and paying for long-term care. Paris: Organization for Economic Cooperation Development (OECD); 2011.

[5] Long-term care insurance. Seoul: Korea National Health Insurance Corporation; 2013.

[6] Sunwoo D. The present situation and problems of the long-term care insurance in South Korea: from comparative perspectives between South Korea and Japan. 2012. http://www.ipss.go.jp/webj.../WebJournal.../Web%20Journal_Dr%20Sunwo.pdf. Accessed 20 January 2015.

[7] Long-term care insurance beneficiaries. Seoul: Korean National Health Insurance Corporation; 2012.

[8] Kim JW, Choi YJ. Farewell to old legacies? The introduction of long-term care insurance in South Korea. Aging Society. 2012; Cambridge University Press. doi:10.107/S0144686X12000335.

[9] Kim EY, Nam ES, Chae YR, Lee HK (2008). Factors affecting the elderly’s preference for utilization of long-term care services based on Andersen’s behavioral model. J Korean Gerontological Soc.

[10] Lee MA (2005). Factors affecting older persons’ expectations of using institutions. J Welfare Aged.

[11] Lee YK (2009). Determinants of long-term care use by elderly. J Korean Gerontological Soc.

[12] Lim JG (2008). A study on factors of elderly residential care service utilization for using decision tree regression. Korean J Soc Welfare.

[13] Lee HS (2013). A study on determinants of a type of using long-term care services for the elderly in Korea. Ph.D. Dissertation.

[14] Park JM (2013). Equity of access to long-term care among the Korean elderly. Health..

[15] Aday LA, Andersen R (1975). Development of indices of access to medical care.

[16] Park JM (1994). The determinants of physician and pharmacist utilization and equity of access under Korean universal health insurance. PhD. Thesis.

[17] Aday LA, Andersen R, Fleming GV (1980). Health care in the U.S.: equitable for whom?.

[18] Park JM (2005). The determinants of long-term care utilization and equity of access to care among older adults in Dong-Ku of Incheon metropolitan city, South Korea. Asia Pac J Public Health.

[19] Aday LA, Andersen R (1981). Equity of access to medical care: a conceptual and empirical overview. Med Care..

[20] Park JM (2003). Equity of access to long-term care among the American elderly. J Public Health.

[21] Park JM (1995). Equity of access under Korean national health insurance: Implications for health care reform.

[22] Lu J, Leung G, Kwon S, Tin KY, Van Doorslaer E, O'Donnell O (2007). Horizontal equity in health care utilization – evidence from three high-income Asian economies. Soc Sci Med.

[23] Shin SM, Kim MJ, Kim ES, Lee HW, Kim HK (2010). Medical aid service overuse assessed by case managers in Korea. J Adv Nurs.

[24] Ahn YH, Ham OK, Kim SH, Park CG (2012). Multilevel analysis of health care service utilization among medical aid beneficiaries in Korea. J Korean Acad Nurs.

[25] Park JM (2014). Health status and health care service utilization in elderly Koreans. Int J Equity Health.

[26] Theobald H (2010). Conditions and challenge of Germany’s long-term care insurance: care policies and inequalities based on gender, socio-economic class and ethnicity. J Asian Women’s Stud.

[27] Jeon H (2007). A comparative study of Korea’s long-term care program. Thesis.

[28] Rodrigues R, Schmidt A (2010). Paying for long-term care.

[29] Sato M, Hashimoto H, Tamiya N, Yano E (2006). The effect of a subsidy policy on the utilization of community care services under a public long-term care insurance program in rural Japan. Health Policy.

[30] National Center for Health Statistics. Vital and health statistics: current estimates from the national health interview survey 1988. ftp://ftp.cdc.gov/pub/health_statistics/nchs/Dataset_Documentation/NHIS/1988/NHISCORE.pdf. Accessed 25 February 2015.

[31] Peabody JW, Rahman MO, Gertler PJ, Mann J, Farley DO, Carter G (1999). Policy and health: implications for development in Asia.

